# The effect of maternal pre-pregnancy body mass index and gestational weight gain on behavioural outcomes in term normal birth weight children: UK birth cohort study

**DOI:** 10.1192/j.eurpsy.2023.1501

**Published:** 2023-07-19

**Authors:** B. A. Dachew, A. A. Adane, R. Alati

**Affiliations:** 1 School of Population Health, Curtin University; 2 Ngangk Yira Institute for Change, Murdoch University, Perth, Australia

## Abstract

**Introduction:**

Existing evidence in the association between maternal pregnancy and pre-pregnancy weight and behavioural outcomes in children.

**Objectives:**

This study aimed to examine these associations at six developmental time-points between ages 3 and 16.

**Methods:**

We used data from the Avon Longitudinal Study of Parents and Children (ALSPAC), an ongoing population-based longitudinal pregnancy cohort study in Bristol, United Kingdom (UK). Data on behavioural outcomes were measured at ages 3.5, 7, 9, 11 and 16 years using the Strengths and Difficulties Questionnaire (SDQ). Over 7960 (at 3.5 years of age) and 4400 (at 16 years of age) mother-child pairs were included in the final analysis. Logistic regression analyses were used to examine the associations.

**Results:**

Pre-pregnancy BMI and gestational weight gain were associated with total behavioural difficulties in children across all age groups. In separate analyses using each SDQ subscale, however, we found that pre-pregnancy underweight was associated with emotional problems at ages 7 (OR = 1.66, 95% CI; 1.20 – 2.29), 11 (OR = 1.49, 95% CI; 1.02 – 2.18) and 16 (OR = 1.74, 95% CI; 1.16 – 2.60) years and hyperactivity/inattention problems at age 16 (OR = 1.96, 95% CI; 1.27 – 3.05). We also found an association between guideline-discordant gestational weight gain and peer relationship problems at age 9 and pro-social behaviour at ages 9 and 11.

**Conclusions:**

Our findings highlight that pre-pregnancy underweight than overweight, obesity or gestational weight gain may influence the emotional health of children and adolescents.

**Disclosure of Interest:**

None Declared

